# Designing for a Healthier Indore, India: Participatory Systems Mapping

**DOI:** 10.1007/s11524-022-00653-3

**Published:** 2022-07-18

**Authors:** Alsa Bakhtawar, Damodar Bachani, Karen Grattan, Bailey Goldman, Neeraj Mishra, Amanda Pomeroy-Stevens

**Affiliations:** 1 Building Healthy Cities Project, John Snow India Private Limited, New Delhi, India; 2 Engaging Inquiry LLC, Durham, NC USA; 3 Building Healthy Cities Project, JSI Research & Training Institute, Inc, Arlington, VA USA

**Keywords:** Systems thinking, Urban health, Healthy cities, Smart Cities

## Abstract

In Indore, India, BHC engaged 247 multi-sector stakeholders through a systems mapping approach to gather qualitative data across three workshops and four citizen town halls from 2018 to 2020. These data were synthesized with results from BHC’s 18 other city activities to build a systems map and identify high-impact areas for engagement. Contextual findings showed a tension at the heart of Indore’s growth—Indore’s great success as a city has spurred rapid population growth. This growth creates pressure on municipal systems as population outpaces service delivery capacity. This is central to the systems map that BHC developed and is expanded upon through additional patterns that fall within four main domains: (1) leadership, governance, and financing; (2) essential service delivery and workforce; (3) information systems; and (4) community infrastructure and education. Stakeholders found three key leverage opportunities within this context that, if included in every action, could help overcome barriers. These opportunities are: (1) improving data quality, use, and integration; (2) supporting accountability to, and enforcement of, policies and regulations; and (3) increasing community engagement. Brought together through a better understanding of the key patterns driving system behavior from the context map and leverage opportunities, BHC was able to co-create, with stakeholders, seven “coherent actions” to move Indore to a healthier, more equitable state. When COVID-19 regulations ease, BHC and city officials will reconvene to finalize an implementation plan for these actions.

## Introduction

Planning for a healthy city is complex. There are many competing forces within an urban system, and these interplay with existing disease burdens and health risks. In addition, multiple levels and sectors must communicate in order to make improvements in the system. The US Agency for International Development (USAID)–funded Building Healthy Cities project (BHC) has hypothesized that a systems thinking approach is a useful method for better defining healthy city planning. BHC partnered with four Asian Smart Cities to test this approach. This paper explores BHC’s systems thinking approach in one project partner city, Indore, India.

Indore, known as central India’s commercial capital, is the largest, most populous city in the state of Madhya Pradesh. Indore was one of the first cities in India to be included in the Prime Minister’s flagship Smart Cities Mission in 2015. It is a fast-growing urban center with a population of just under 2 million in 2014 and a 10-year growth rate of about 3% [1]. It is no surprise that Indore citizens have great pride in their city [2, 3]. Citizens’ pride, knowledge, and engagement are powerful assets that can further transform the city. As its population has grown, so has the number of slum dwellers, who are estimated to make up 30% of the total city population [4]. These factors have made Indore diverse in terms of housing, income level, and health needs.

Food safety is a priority issue in India, and as a result the country enacted the Food Safety and Standard Act, 2006 and set up the Food Safety and Standards Authority of India (FSSAI) to monitor food safety [5]. Indore is known for its delicious street food, and the city has taken steps to improve the quality and safety of food served in its popular food streets; in fact, Indore is the first city in Madhya Pradesh to receive the coveted “Clean Street Food Hub” tag, certified by FSSAI, for two street food hubs. One of these, Chappan Dukan, has received the tag twice, in 2019 and 2021 [6]. However, high consumption of calorically dense, nutrient-poor foods contributes to an increase in obesity and lifestyle diseases like diabetes in Indore. This overlies an existing burden of micronutrient deficiencies; across urban Indore, 24% of women and 21% of men are overweight, and 46% of women are anemic [7]. Other considerations relevant to healthcare include inadequate antenatal care coverage (61% of women had four or more antenatal care visits), stunting of children under 5 years old (39%), and low levels of cancer screenings (< 1%) [8, 9].

Indore’s physical environment is also rapidly changing. In 2021, Indore was recognized by the Clean India Mission as the cleanest city in India for the fifth consecutive year, based on major improvements to waste management and sanitation [10]. In 2021, it was also recognized as the first Water Plus city in India [3], due to improvements in water access, public toilets, and wastewater management. While the city has experienced modest improvements in air, noise, and water pollution, concerns remain about environmental risk factors for diseases [11]. Results from a participatory research study conducted by BHC in eight informal settlements in Indore suggested that the biggest environmental health concerns in urban poor communities were: access to clean water, inadequate wastewater management leading to clogging of drains, use of biofuels, and risk of vector and water-borne diseases [12].

The city has made improvements to its information systems by developing an Integrated Command and Control Centre (ICCC), a central system that consolidates data from a range of sectors and services including traffic and solid waste management. The ICCC also integrates the Indore 311 app and CM Helpline citizen reporting systems, which allow citizens to submit complaints and assistance requests directly to the relevant city departments. However, a lack of collaboration and sharing of data across sectors remains a persistent issue [13].

Considering the multi-sectorality of these issues, BHC sought an approach that could address two important questions, informed by findings from our baseline assessments [13–15]: (1) How can policymakers and service providers who govern the urban development of the city more thoroughly consider the full health implications of their work; and (2) by what means can BHC facilitate more, and more sustained, multi-sector collaboration in the service of healthy urban planning? In the case of Indore, Indore Smart City Development Limited (ISCDL) had already prepared a detailed master plan that focused on solutions for water supply, solid waste management, sewerage, social infrastructure, and traffic flow, and they were interested in support to more fully incorporate public and environmental health issues [16].

While the benefits of multi-sector collaboration are fairly well accepted, there are common barriers and challenges in operationalizing the concept [17]. BHC, in partnership with ISCDL, used dynamic systems mapping to underpin a systems thinking approach, which we hypothesized would be an effective tool to bring city planners and administrators together with citizens and civil society organizations to create traceable, transparent, and collaborative policy initiatives.

BHC is not the first project in the Indian context to use systems thinking or to support prioritization efforts for Smart Cities. Rana et al. (2019) used a fuzzy Analytic Hierarchy Process to identify key barriers across all Indian Smart Cities and prioritize them for policy purposes. Within the 31 barriers found, the authors prioritized those related to “governance,” with the top five sub-barriers for this category being: political instability; lack of cooperation and coordination between a city’s operational networks; unclear information technology management vision; poor private–public participation; and lack of trust between the governed and government [18].

Several papers have attempted a formal conceptual definition of “Smart Cities” from a systems thinking view, but stopped at the conceptualization phase [19, 20]. We found two studies in the Indian context applying some form of systems thinking or mapping to questions of health (one on healthcare systems in Tumkur District and another on urban vaccination in Kerala), but neither applied those findings to policy or planning [21, 22]. To our knowledge, BHC is the first project of its kind in India that has demonstrated the utility of systems thinking, and specifically systems mapping, as an evidence-based policy and planning approach to develop smart and healthy cities in India.

Looking to Indore specifically, BHC builds upon earlier efforts implemented since 2010 aiming to incorporate other sectors into urban planning. In the early to mid-2010s, there were efforts to consider health impacts of bus transit which included: a city-wide health impact assessment; community-based climate change adaption activities to bring a more multi-sector viewpoint on how to address water scarcity in particular in urban planning; and continuing efforts by the Urban Health Resource Centre to promote health and well-being within Indore’s informal settlements [23–25].

These efforts were still fragmented and in some cases largely driven by outside forces until Indore won funding from the Smart City Mission in 2016 [26]. This created a central mechanism for coordinating several sectors around a master urban plan that considered health and well-being among the key outcomes. While ISCDL made plans for health-related Smart City activities, when BHC began in 2017, the ISCDL office expressed frustration with finding appropriate mechanisms to spend those funds [4]. Around the same time, the Indore Municipal Corporation had been able to make great multi-sector strides in addressing waste management via the Swachh Bharat campaign [27]. BHC’s value addition to these efforts was to provide a practical multi-sector planning model that could incorporate the community focus of Swachh Bharat and fit into the data systems being developed by ISCDL, while building a platform for regular multi-sector communication and collaboration toward a mutually agreed upon goal of a healthy and livable Smart City for all.

## Methods

Systems mapping is a visual depiction of a system. While these maps can be developed from any combination of qualitative and quantitative data, Indore’s map was developed from primary and secondary qualitative data. BHC used a grounded theory approach to tease out how different key elements of the city (i.e., various government departments, citizens, the environment, infrastructure, data systems, etc.) interacted with each other over a period of time, forming patterns of behavior across the system. It is important to note that the participatory element of this work extends through defining of vision statement, data collection, and data analysis, with stakeholders themselves helping to analyze the data to come to conclusions within the workshops on loops, relationships, opportunities, and actions.

In order to validate the secondary data analysis and collect the primary data, BHC led a series of participatory, multi-sectoral workshops and town hall meetings described in Table 1. For a full description of the underpinnings and processes for this type of mapping, please see Pomeroy-Stevens et al. (2022) in this issue [28].

**Table 1:** Summary of Indore’s systems mapping process

Step	Dates	Source of data used to facilitate workshop	Participants	Data analysis
Defining context	28–29 August 2018	Analysis of baseline assessment data	48 stakeholders	Cause-and-effect analysis to develop casual loops.
Finding leverage	Leverage workshop: 21 June 2019	Context maps	35 stakeholders	Workshop data synthesis. Develop systemic change hypothesis. Visualize on leverage map.
	4 Town Halls: (1) 28 January 2019 (2) 21 February 2019 (3) 14 June 2019 (4) 31 August 2019	Relevant loops from context maps	(1) Women in slums: 30 women (2) Youth: 33 participants (3) Sanitary workers: 53 workers (4) Frontline health workers: 47 participants	Share some of the loops from the context map to seek feedback of various community members.
Creating action	13–14 February 2020	Context maps and leverage hypotheses	29 stakeholders	Synthesize responses to identify key themes proposed by the participants. Use BHC triangulation to complete action plan.

Source: For more details on these workshops, please see [29–31]

In the first step of analysis, the authors used Word and Excel to re-analyze qualitative data collected during three baseline assessments [13–15] to identify key barriers and enablers relating to the themes uncovered during the grounded theory analysis [32]. These were presented to stakeholders at the first workshop, who first received an introduction to systems analysis and discussed the vision statement, which is meant to provide a common goal for all participants across the mapping process. They then participated in focus group discussions where BHC used semi-structured questionnaires to facilitate discussion on the causal relationships at play within the system across these themes. Each subsequent workshop used similar facilitated, participatory analysis to develop the next level of findings, which we then built into the visual maps, similar to chapters of a report. After each workshop, the authors worked together to test the logic of the relationships within and across themes in order to build the causal loop diagrams in Kumu software, which was the primary vehicle for synthesizing the results [33]. Readers will find links to the Indore maps throughout this article and are encouraged to review them for more detail. Because these maps are meant to be living depictions of the system, as BHC received new evidence or completed other relevant studies, those findings were reviewed against the maps and in many cases incorporated into the narratives as citations.

Sampling of participants was done purposively to achieve representation from all key sectors, defined from the baseline data collection and our discussions with city leadership during the establishment of the project. Total sample sizes aimed to reach saturation on the qualitative themes, but as no quantitative data were collected, they were not designed to be statistically representative of the population. In some cases, we did not reach saturation on a particular theme within the workshop, and in those cases we followed up with individuals for interviews for further information, and in some cases we were able to draw from our other research activities to fill gaps [34–36]. This was true for some themes relating to food safety, traffic and road safety, and waste management, where officials attended some but not all systems approach workshops.

In total, BHC engaged 247 stakeholders from 2018 to 2020 during the Indore systems mapping process. This number includes the 38 city officials and workers from different government departments including: Health and Family Welfare (including National Health Mission), Women and Child Development, Education, Indore Municipal Corporation, ISCDL, and Madhya Pradesh Pollution Control Board.

## Results

BHC and stakeholders built off Indore’s Smart City goal statement from 2016, reframing it to integrate healthy city goals [37]*.* This resulted in a vision statement for Indore’s systems practice: “*Indore, a healthy and livable Smart City for all, with healthy citizens, clean air, and equitable access to health care and waste management services.*” This vision statement was used to guide the remaining steps of the systems mapping process.

**Fig. 1:**
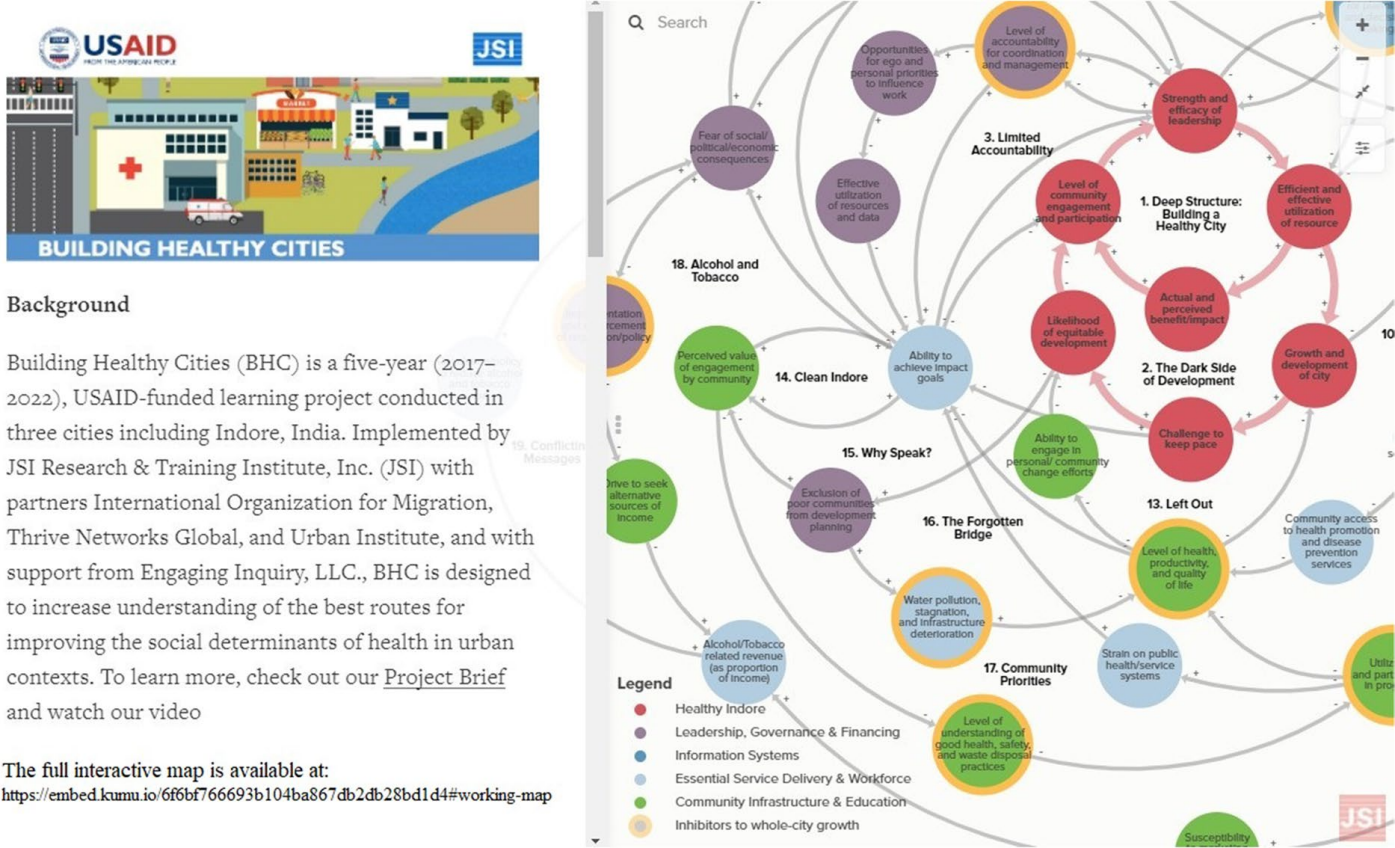
Tension at the center of Indore’s success

The analysis uncovered a central tension at the heart of Indore’s development. This tension comes from Indore’s great success as a city, spurring rapid growth as people from around the country seek opportunities there, and the pressure placed on municipal systems when population outpaces service delivery capacity. Figure 1 visualizes this tension and its interconnections with the other elements of the systems.

The full context map, accessible here (https://embed.kumu.io/6f6bf766693b104ba867db2db28bd1d4), encompasses 19 loops. Further analysis of the thematic elements across loops presented four domains. These domains are defined as follows:

- Leadership, governance, and financing: This domain holds loops related to accountability and management within governance structures; defining, developing, and enforcing policies, procedures, rules, guidelines, etc.; and continual monitoring for proper implementation. It also reflects the need for efficient use of, and accountability over, those resources. Without these structures in place and enforced, the data suggest there is an opportunity for ego, personal priorities, and fear of social/political consequences to negatively influence the way programs are implemented, evaluated, and funded. Creating opportunities to link sectors, data, and citizen feedback into leadership and governance of projects will increase the ability of programs to achieve their desired impact and reduce the risk to leaders of programs that are poorly received by citizens. The evidence that underlies the map and this domain supports the fact that Indore has shown tremendous leadership and governance in the successful implementation of the Clean India Mission in the city, and this can be leveraged to make positive gains in this domain.
- Information systems: Information systems are an integrated set of components for collecting, storing, and processing data for sharing information. The purpose of these information systems is to transform raw data into quality evidence and knowledge for decision-making. Accurately entered, up-to-date, and complete data was recognized as a challenge in the current system. BHC found in its 2018 baseline assessment of data use and access that while information systems that support city services across seven health-related sectors were slowly digitizing as part of efforts such as Aadhaar, Amrut, and the National Health Mission, not all of these systems were designed to give cities usable information or to be inter-operable with each other [13]. Workforce limitations can also affect access and use of data. Factors identified by interviewees and workshop participants were fear of consequences for not meeting goals, limited staff capacity to analyze and use data, and low expectations for external data reporting. Yet participants acknowledged the essential role data plays in program planning, resource allocation, and monitoring and evaluation of programs. Limited multi-sector collaboration may affect the ability of the city administration to effectively allocate resources for sectors where these are needed most. Conversely, lack of access to multi-sector data also limits more multi-sector collaboration. This adds to the strain of growth on the system and limits the potential for whole-city healthy development. A real bright spot in this area is the effort by city leadership to develop the ICCC to link and integrate data systems across sectors. The inclusion of health and healthcare-related data systems would be a step forward to assess the effectiveness of Indore’s developmental initiatives.
- Essential service delivery and workforce: Any planning by the city administration needs to ensure equitable access to the basic needs of the citizens. Essential services are those that are necessary for citizens to not only feel safe and survive, but also experience quality of life and productivity. Participant feedback from several sectors provided evidence that limited financial resources for essential services create shortages in human resources (difficulty with hiring, retention, and capacity building) and barriers to procuring equipment and developing infrastructure. When staff and facilities are not able to operate to their highest potential, the quality of care suffers and both clients and workers may opt to avoid the public sector and instead seek care or employment in the private sector. In the health sector in particular, citizen health-seeking behavior has steadily shifted to the private sector for years. Stories provided by participants in both the workshops and the town halls suggest that private sector providers mainly focus on acute medical care (“treatment”) alone and miss opportunities for health promotion and preventive care. Similarly, in waste management, while garbage collection has been a priority, low investment in local infrastructure resulted in backed up or broken drains that increased risk of illness and injury, and barriers to safe transport.
- Community infrastructure and education: Most often, program and policy planning is conducted by policymakers and does not actively involve beneficiaries in the process. The ability to engage the population in healthy development efforts is a theme that resonated strongly in our qualitative data as the key to achieving program impact goals and enabling strong leadership. However, even when policymakers do reach out to the community, if community members are regularly unable to meet basic needs, access critical programs and information, or are shown that their participation is not valuable, their trust and willingness to engage with policymakers and planners decrease. The data in the loops in this domain indicate that when programs are implemented without community input, they are unlikely to be successful and, in addition to wasting time and resources, high-risk populations will continue to be excluded from the benefits of city development. This leads to increasing inequities in access to good health, well-being, and economic productivity, which undermines the growth and development of the city as a whole.

Based on the evidence gathered in the Context Map, BHC identified key obstacles that should be addressed and opportunities to be maximized in the next few years to achieve the goal statement of a healthier Indore.

### Theory of Change

Forty percent of sectors and organizations that were represented at the Context Workshop returned for the leverage phase. Stakeholders were asked to flag areas where: change was unlikely (“frozen” areas), areas ripe for change (“energy” areas), and areas where a shift would create ripples of impact (“ripple” areas). Participants were facilitated through group analysis of the flags to identify areas with the greatest potential to shift important behaviors or relationships in the system. Based on this analysis, stakeholders found three key leverage opportunities within this context that, if included in every action, could overcome barriers and strengthen the system. The three opportunities are described in Table 2 below and illustrated in the interactive Leverage Map (https://embed.kumu.io/626e210478e391ccdff94716f07fc9a9).

**Table 2:** Leverage opportunities for Healthy Indore system

Leverage opportunity	Description
Improving data quality, use, and integration	If the city improves the mechanisms for cross-sectoral data sharing, increases training and incentives for data collection and management staff, and develops uniform data collection tools and protocol, then the anticipated impact will be: higher quality data, improved data utilization, and increased impact of policy and program design.
Supporting accountability to and enforcement of policies and regulations	If the city increases structures of accountability for effective coordination and management of programs and implements enforcement measures in support of policies and regulations, then the level of compliance and participation (within public and private sectors, as well as at the community level) will increase. The anticipated impact of this change in the system is that the level of deterioration and health concerns within existing infrastructure will go down and the likelihood that new development will be designed equitably will increase.
Increasing community engagement	If the city designs programs based on community need and input (specifically marginalized populations), increases staff resources and training, and demonstrates strong, trustworthy leadership, then the anticipated impact will be: increased access and utilization of health promotion/disease prevention programs, improved health and productivity of the population, and a stronger system of care.

### Theory of Action

More than 50% of the stakeholders from the Leverage Workshop returned for the action planning phase. The aim of this final workshop was to develop a set of suggested actions to move Indore toward its vision statement. This work resulted in four aligned and mutually beneficial actions to move the city to a healthier state. Three additional action areas were developed by cross-referencing with the research and evidence generated over the life of the project. The final seven coherent action areas are described in Table 3 below. These are not meant to be comprehensive, but rather cover the majority of obstacles and opportunities using the three leverage opportunities, and rapidly build on current energy. Because they were developed via participatory inquiry, they are bound by what stakeholders see as within the realm of possibility for their city.

**Table 3:** Proposed Indore Healthy City actions

How might we…	Proposed coherent action summary
Ensure access to healthy food for every Indorean?	The purpose of this action is to strengthen the food systems in Indore to make healthy and standard food available and accessible to all sections of society. This would happen by: organizing awareness of citizens through media; building the capacity of city officers dealing with food safety, owners of food establishments, and vendors; and strengthening compliance to the prevailing laws. This would create not just healthier people through decreased malnutrition and food-borne diseases, but also opportunities to improve livelihoods in the city.
Ensure awareness about healthy food and hygiene among food handlers and citizens?	
Avoid making air quality worse as Indore grows?	The purpose of this activity is to create better air quality and livability in Indore via citizen participation. This would happen by building the knowledge of the community on causes and consequences of air pollution and involving them (and using their feedback) in environmental planning and management. This model realizes that environmental management is not just the responsibility of the government; individuals, communities, civil societies, organizations, etc. are also responsible.
Make citizens more accountable regarding their surroundings?	
Make citizens aware about the health impacts of air pollution?	
Make it easier to use data to support health in Indore?	The purpose of this activity is to improve the quality, timeliness, and usability of data across sectors and systems relating to maternal and child health. This would include building the capacity of frontline health workers and use of existing technology and resources such as the Auxiliary Nurse Midwives Online (ANMol) app and Integrated Control and Command Centre. This would create a trained cohort of staff, improve data accuracy, reduce the burden on frontline health workers, and ensure accountability of health workers to complete assigned tasks.
Encourage and build capacity of healthcare workers to collect and upload real time monitoring data?	
Strengthen the existing data systems?	
Grow a healthier next generation of citizens?	The purpose of this activity is to build Indore into a child-friendly city by keeping the focus on health and living environments of children through a bottom-up approach, active community participation, and multi-sector engagement. This would create a safe, healthy, and livable environment for children coming from all sections of society.
Develop pedestrian- and child-friendly pathways?	
Encourage children to adopt health-promoting behaviors?	
Grow our transport infrastructure capacity while also reducing our risk of noncommunicable diseases?	The purpose of this action is to create a diverse and equitable transport infrastructure in the city from which people from all sections can benefit. This would decrease transport barriers, lower air pollution, increase road safety, and reduce household transport costs.
Unclog waterways and drains while also addressing the need for more job opportunities in informal settlements?	The purpose of this action is to build livelihoods while at the same time reducing waste streams and free waste in neighborhoods in Indore. This would build from Indore’s strengths in waste management to lift many households out of the lowest level of poverty while also reducing the methane, CO_2_, and other climate-changing gases coming from solid waste.
Foster accountability, communication, and coordination and make policy decisions and processes more transparent and participatory?	The purpose of this action is to support a Healthy Indore secretariat using the existing Smart Health Working Group. This would provide a sustainable funding support mechanism to continue progress toward a healthier Indore.
Sustain a whole-city Healthy Indore effort?	

## Discussion

The final outputs of this mapping process helped BHC develop a multi-sector “Healthy Indore Action Plan” that breaks the developed actions into practical next steps including sector-level sub-actions, cost estimates, and roles and responsibilities. BHC facilitated a series of multi-sector consultations to finalize the plan in August 2021 and launched the completed plan with the city in March 2022. BHC also funded a pilot of the Action Plan, called Kaya Kalp, in two neighborhoods. BHC used settlement-level data collected in a participatory fashion in 2020 to help define the needs-based implementation approach in the pilot, which also included citizens ranking actions they would like to see implemented in their neighborhood [38]. This pilot is important for testing the equity-based application of the Action Plan, which means each neighborhood may receive some sub-set of actions based on needs and demands.

Systems thinking is a natural fit for healthy urban planning; it brings together diverse stakeholders to identify and implement coordinated actions towards whole-system change. These stakeholders then have a practical tool—the systems map. This work was complemented by other systems strengthening activities, including the formation of the Multi-Sector Smart Health Working Group. The ownership of this group lies with ISCDL, with BHC acting as facilitator. The group is chaired by the CEO of ISCDL, co-chaired by the Chief Medical and Health Officer of the Department of Health, and consists of representatives from different government departments and organizations, development partners, academia, etc. While the formation of this group was separate from the systems mapping, there is significant overlap in membership, and it provides a critical venue for regular discussion and operationalization of the mapping results and Action Plan.

In addition to positive feedback from Indore city leadership on the systems mapping process (see Bachani et al. in this issue), BHC has received additional feedback and interest in use of systems mapping and systems thinking from the Smart Cities Mission (SCM) in the Ministry of Housing and Urban Affairs [39]. The Mission Director of SCM has been working on various initiatives to improve urban health outcomes in Smart Cities and has found systems thinking to be a very useful way to organize priorities and deal with complexity. BHC was asked to work with a core team at SCM to share resources on systems thinking, advise on a set of multi-sector indicators for healthy cities, and develop a training curriculum on systems mapping.

Despite the success of the BHC-led systems approach in Indore, there were certain limitations that the project faced, including frequent turnover in high-level governmental positions and difficulty engaging service providers due to busy schedules. Restrictions caused by COVID-19 shortly after the action planning phase also made it difficult to safely organize remaining sector consultations as planned. BHC adapted our approach by focusing on one-on-one meetings, but we were not able to re-convene the multi-sector group to share action results until mid-2021. There was also a considerable time gap between workshops on theory of context, theory of change, and theory of action. As BHC has refined this approach in our other project cities, we have found ways to shorten this timeline that has helped to keep stakeholders more fully engaged.

Other cities can apply learning from BHC’s systems work in one of the fastest-growing and rapidly changing Smart Cities in India. A systems approach at the local (city or district) level conforms with decentralized planning and implementation to support recent movements towards bottom-up approaches to planning and funding key programs (e.g., National Health Mission). Broad policies advocated by national and state governments allow flexibility in operations and funding to meet local needs and demands (e.g., a special vehicle under Smart City Mission). The systems approach provides actionable tools and methods for identifying the best possible actions and investment opportunities to utilize available resources in the most cost-effective manner.

## Conclusion

BHC’s effective collaboration with Indore city administration, partners, and other stakeholders and their active engagement in various workshops on systems thinking led to development of a draft Action Plan to make the city a healthier place. The forum for multi-sectoral coordination set up under BHC will be continued by ISCDL to ensure effective implementation of the Action Plan. The Indore systems thinking model is also likely to be adopted by peer Smart Cities in India in the coming years, in order to work towards achieving goals set up under SDG 2030**.**

## Data Availability

The resulting Context (https://embed.kumu.io/6f6bf766693b104ba867db2db28bd1d4) and Leverage Maps (https://embed.kumu.io/626e210478e391ccdff94716f07fc9a9) are available on the open-source platform Kumu.
